# Adverse Effects of Pfizer (BioNTech), Oxford-AstraZeneca (ChAdOx1 CoV-19), and Moderna COVID-19 Vaccines among the Adult Population in Saudi Arabia: A Cross-Sectional Study

**DOI:** 10.3390/vaccines11020231

**Published:** 2023-01-20

**Authors:** Jehad Aldali, Sultan Ayoub Meo, Thamir Al-Khlaiwi

**Affiliations:** 1 Department of Pathology, Faculty of Medicine, Imam Mohammad Ibn Saud Islamic University, Riyadh 13317, Saudi Arabia; 2 Department of Physiology, College of Medicine, King Saud University, Riyadh 11461, Saudi Arabia

**Keywords:** SARS-COV-2, adverse effects, Pfizer-BioNTech, Oxford–AstraZeneca (ChAdOx1 CoV-19), Moderna

## Abstract

The Severe Acute Respiratory Syndrome Coronavirus-2 (SARS-CoV-2) outbreak has caused massive damage to the global healthcare system and economy. To compete with the SARS-COV-2 pandemic, several vaccines have been proposed to immunize the population. The present study aimed to investigate the adverse effects following the three doses of COVID-19 vaccination, Pfizer (BioNTech), (Oxford-AstraZeneca (ChAdOx1 CoV-19), and Moderna among the adult population in the Eastern province of Saudi Arabia. In this study, the total number of participants were 426, among them 277 (65%) were females and 149 (35%) were males. An online survey using Google forms in the English language and translated into the Arabic language was used to record the information. The questionnaire was distributed to participants who received either Pfizer-BioNTech, Oxford-AstraZeneca or Moderna vaccines. The general characteristics of participants were obtained, alongside an evaluation of the vaccination’s adverse effects. The results revealed that Pfizer-BioNTech COVID-19 vaccines caused significantly less adverse effects than Oxford–AstraZeneca (ChAdOx1) and Moderna (*p* < 0.001), and females experienced more adverse effects after vaccination compared to males. Injection site pain was the most common adverse event among the participants (60.6%), followed by fatigue, headache, and pain (43.9%), muscle and joint pain (32.4%), increased body temperature and shivering (24.2%). In addition, the group of individuals under the age of sixty was more likely to experience side effects than the participants with other age groups. All three vaccines, Pfizer-BioNTech, Oxford–AstraZeneca (ChAdOx1 CoV-19) and Moderna, cause post-vaccinal adverse effects; however, Moderna and Oxford–AstraZeneca (ChAdOx1) causes adverse effects more frequently than the Pfizer-BioNTech.

## 1. Introduction

The Severe Acute Respiratory Syndrome Coronavirus 2 (SARS-CoV-2), also called COVID-19, pandemic, since its outbreak has caused massive damage and result in challenges to all countries around the world [1]. On 23 December 2022, the disease had infected 651,918,402 people and caused 6,656,601 deaths, with a case fatality rate of 1.02% [2].

Since December 2019, the world has witnessed the various waves of the deadly pandemic cause various clinical manifestations in the general population [3,4]. SARS-CoV-2 is a highly contagious disease and spreads with mutable prevalence and mortality outbreak trends [1]. Worldwide health officials and policymakers took highly preventive measures to combat this deadly pandemic situation. These precautions included complete lockdowns, face masks, social distancing, and extensive vaccination campaigns to prevent and eradicate the disease [5]. In 2020, several clinical trials and research studies related to COVID-19 vaccines were conducted to develop an appropriate vaccine to fight against the pandemic. The vaccines included the Pfizer-BioNTech messenger RNA (mRNA) vaccine (BNT162b2), Moderna, Johnson & Johnson vaccines, Sinopharm and Oxford–AstraZeneca (ChAdOx1 nCoV-19) [5,6,7,8].

The Saudi Food and Drug Authority (SFDA) approved two vaccine schedules for the coronavirus disease (COVID-19). Among them is an RNA-based vaccine from Pfizer on the 17th of December 2020 and a lipid nanoparticle-encapsulated vaccine from AstraZeneca that encodes the spike protein antigen from SARS-CoV-2 in February 2021 [8,9]. The Moderna vaccine was authorized in the Kingdom of Saudi Arabia on the 16th of July 2021 [10]. The effectiveness of SARS-CoV-2 vaccines has led to a significant reduction in mortality [10]. Following recent evidence that the COVID-19 vaccine may have resulted in weakened immunity, additional doses and boosts have been approved and distributed. In addition, the Centers for Disease Control and Prevention (CDC) recommends the COVID-19 booster vaccine for Pfizer-BioNTech and Modena recipients to those who have completed their initial series for at least six months.

The most common type of adverse events are systematic and local side effects. The systemic side effects include fatigue, headaches, muscle aches, chills, and fever, and local side effects include pain at the injection site leading to discomfort, redness, and swelling [11,12,13]. There is truly little knowledge about what happens following post-vaccination in the general population. The public’s belief in the safety of vaccines will increase, the fear of diverse types of vaccines will be reduced, and the vaccination process against COVID-19 will be accelerated. This study aimed to evaluate the adverse effects following the three doses of COVID-19 vaccination, Pfizer (BioNTech), (Oxford–AstraZeneca (ChAdOx1 CoV-19), and Moderna among the adult population in the Eastern province of Saudi Arabia.

## 2. Subjects and Methods

### 2.1. Study Design and Settings

This cross-sectional study was conducted in the Department of Pathology, Faculty of Medicine, Imam Mohammad Ibn Saud Islamic University, Riyadh, Saudi Arabia.

### 2.2. Study Participants

The study participants were the general population residing in the Eastern province of Saudi Arabia. The study participants were students, teachers, clerical staff, technicians, shopkeepers, and healthcare workers who had received three doses of the COVID-19 vaccination, Pfizer (BioNTech), (Oxford–AstraZeneca (ChAdOx1 CoV-19), and Moderna. The research team visited various places including schools, universities, hospitals, student councils and health institutes in the eastern region of Saudi Arabia to collect the contact details of the participants.

### 2.3. Inclusion and Exclusion Criteria

We included participants who had received three doses of the COVID-19 vaccination, Pfizer (BioNTech), (Oxford–AstraZeneca (ChAdOx1 CoV-19), and Moderna. The exclusion criteria included those who had not received three doses or had been vaccinated by any other vaccine besides Pfizer (BioNTech), (Oxford–AstraZeneca (ChAdOx1 CoV-19), and Moderna, or who had not been vaccinated for COVID-19.

### 2.4. Sample Size Calculation

The study sample size was calculated following the study protocol [14] and using the “Raosoft” power calculator [15]. The Eastern province of Saudi Arabia regional authorities aimed to vaccinate 70% of the population, approximately 1,250,000 individuals, and a sample size of 385 participants was sufficient to achieve 95% confidence with a 5% margin of error. However, in the present study, 426 have participated.

### 2.5. Study Questionnaire Development

An English-language, translated to Arabic, self-administered, validated online web-based questionnaire was developed and circulated via a link survey to different platforms on social media and other mailing platforms, such as email and WhatsApp [16,17]. The survey was distributed to participants who received either Pfizer-BioNTech, Oxford-AstraZeneca or Moderna vaccines in the Eastern Region of Saudi Arabia from the 10 May 2022 to the 10 August 2022.

It was initially distributed to a pilot sample consisting of 06 faculty members to countercheck the validity of the questionnaire and for any technical concerns. We attempted to make the survey as brief and to the point as possible. We explained that the information provided would be only utilized for research purposes because the participants were not identified and had the right to withdraw at any stage.

The questionnaire had an introductory paragraph explaining the nature and objectives of the study and the voluntary and anonymous nature of participation. The first section of the online survey focused on participant demographic data, including age, gender, smoker or non-smoker, current health status, and SARS-CoV-2 infection status. The second section focused on the information about the COVID-19 vaccine, the type of vaccine, the date of the vaccination (first, second, or third dosage), the side effects following the vaccination, the severity of the side effects and the length of the side effect. Participants were asked to describe their symptoms following vaccination, including headache, tiredness, pain at the site of injection, muscle and joint pain, temperature, shivering, dizziness, vomiting, congestion, breathing difficulty, menstrual disorder, skin itching or other side effects. Then the severity was assessed as mild, moderate, or severe. Participants were also asked how long symptoms lasted after they first appeared. Finally, participants were asked if any analgesics were consumed and whether they helped relieve symptoms. All participants who chose not to participate or did not receive three doses of the COVID-19 vaccine were excluded.

### 2.6. Ethical Statement 

The Institutional Review Board, College of Medicine, Imam Muhammad bin Saud Islamic University approved the study (Ref. 271-2022).

### 2.7. Statistical Analysis

Statistical analysis was carried out by using the software Statistical Package for Social Sciences (IBM-SPSS) version 25. The descriptive statistics were calculated in the form of frequencies and percentages to describe the study samples, vaccines, and adverse events. The Kolmogorov–Smirnov and Shapiro–Wilk tests were used to check for data normality or data distribution. After this, significant associations of the variables with adverse events of the vaccines, and cross-tabulation with the Chi-square method were used. Moreover, a general linear model (multivariate analysis of variance- MANOVA) was carried out to inspect significant differences in adverse events.

## 3. Results

### 3.1. Demographics

In this study, four hundred and thirty-sex participants completed the survey. However, ten subjects were excluded because they did not meet the inclusion criteria. Therefore, the total number of participants were 426. The majority of participants were from the age group between 41 and 60 years old 149 (35%); 18 and 25 years 113 (26.5%); and more than >60 years of age were 28 (6.6%), with a mean age 36.4 years. In terms of nationalities, 401 (94.1%) were Saudi, while the remaining 25 (5.9%) were non-Saudi. The majority of participants were females 277 (65%), and the males were 149 (35%). Table 1. provides a summary of the participants’ general demographic characteristics and medical history, with 331 (77.7%) participants claiming no clinical background; 95 participants (22.3%) were confirmed to have a chronic disease. The survey also questioned participants’ smoking habits, which reported 362 (85%) were non-smokers and 64 (15%) were smokers.

### 3.2. Adverse Effects of COVID-19 Vaccinations

The most common adverse effects of COVID-19 vaccines from AstraZeneca (ChAdOx1), Moderna, and Pfizer-BioNTech following the first, second, and third doses are listed in Table 2, followed by the rare side effects following the first, second and third dosage of COVID-19 vaccination (Table 3). Within the three doses, the most common vaccine taken by the participants was Pfizer-BioNTech (approximately 80%), followed by Oxford-AstraZeneca (ChAdOx1), and then Moderna. However, in the third dosage, it was found that Moderna was the second most common booster vaccination taken by subjects compared to the Oxford-AstraZeneca vaccination.

The onset of symptomatic side effects was primarily identified on the first or second day following the vaccination in all three dosages. Approximately 70% of participants suffered from adverse events in both the first and second doses, and only 60% suffered from side effects following the vaccination from the third booster vaccination. The most common side effects following the three dosages were pain at the site of injection, followed by fatigue and headache, and muscle and joint pain (Table 2). It was found that 90% of participants did not take any analgesics due to the side effects encountered following the first vaccination. However, 45% to 52% of participants have taken some medications to reduce the severity of the side effects.

The occasional adverse effects including vomiting, breathing congestion, skin rashes, and dropped-sugar levels were also reported following all the three dosages. However, these rare side effects were less than 3.5% among the participants. After the first vaccination, skin itching, and rash were encountered in approximately 4% of the participants. The severity of the adverse effects was experienced more in females compared to males (*p* < 0.001). It was also identified that the severity of symptoms after the first dose, second dose, or the third dose was more frequent in females compared to males.

### 3.3. Severity of Symptoms According to the Type of COVID-19 Vaccines

The study also looked at the severity of adverse effects that were assessed by participants three days after receiving the vaccination. Calculating the differences using non-parametric statistics using the Kruskal–Wallis Test and Tests of Normality because the number of individuals in some groups < 30.

Table 4, Table 5 and Table 6 provide a summary of the applicants’ general statistically significant differences (*p* < 0.001) in the severity of symptoms after the first dose between those who received the diverse types of vaccines, where the severity of symptoms was higher among those who received the Moderna vaccine then AstraZeneca Oxford more than the Pfizer vaccine. However, there were no significant differences (*p* > 0.05) in the severity of symptoms after the second and third doses between those who received the diverse types of vaccines. We made dimensional comparisons between groups in the severity of symptoms among those who received distinct types of vaccines after the first dose (which showed statistically significant differences in the severity of symptoms) using the Mann–Whitney Test.

## 4. Discussion

The SARS-CoV-2 pandemic, which first appeared in December 2019, continues to spread around the world. The virus causes coronavirus disease and patients may be symptomatic or asymptomatic; however, 20% of affected individuals were at risk of developing moderate to severe disorders with fatal outcomes [18]. The vaccines are the most effective public health intervention because they reduce mortality by preventing and controlling the spread of infectious diseases [19]. Some countries have adopted strict policies of vaccination requirements for COVID-19 into local law to cover both the public and private sectors, requiring travelers to be vaccinated before visiting these countries. Three vaccines that were approved and launched in Saudi Arabia were Pfizer-mRNA BioNTech’s vaccine (BNT162b2), Oxford-vaccine AstraZeneca’s (ChAdOx1 nCoV-19), followed by Moderna Spikevax approval in May 2021 [20].

In this study, most of the adverse effects in the first dosage were 101 (23.7%), with an increased number of no side effects after the second and third vaccines, 134 (31.5%) and 175 (41.1%), respectively, after the vaccine. The total number of people who reported receiving the Pfizer-BNT162b2 vaccine was more than Moderna and Oxford AstraZeneca (ChAdOx1), but the side effects were significantly higher with Moderna and AstraZeneca (ChAdOx1) than Pfizer-BioNTech. It was typical and in line with previous research findings [21,22,23,24].

In the present study, the reported symptoms varied between males and females, as well as between age groups. These facts prove the existence of a relationship between gender and the severity of side effects after vaccination. Females experienced high adverse effects compared to males. The adverse effects that occurred moderately and severely after vaccination were typical and in line with previous research findings; muscle pain, headache, gastrointestinal problems, and fever were among the most reported side effects [23,24]. In females, severity remained high until after four days. The difference in severity levels may be linked to gender-related aspects that were seen by other studies. Studies in the literature claim that women generally have a higher pain threshold than men when considering psychological aspects, such as attitudes toward gender roles, coping mechanisms, or physiological elements such as sex hormones. Moreover, there are more cases of chronic pain in women than in men [23,24]. We investigated the relationship between the severity of reactions after vaccination and the presence of chronic diseases in people who received vaccinations. It was found that the emergence of adverse effects after vaccination was not significantly associated with the prevalence of chronic conditions.

The findings of Al-Hazm et al., [25] who investigated the short-term negative effects of COVID-19 vaccines on Saudi citizens and investigated that the infection rate is higher in females than in males, directly contradicts this finding. When questioned about the drugs they were taking due to minor side effects, most participants who experienced severe side effects required medicine to treat the side effects in the first and second dosage. However, some participants did not require medication at all. When comparing age groups, it was found that people under 60 years of age were more likely to have side effects. More studies are still needed to confirm these observations [25].

In public, concerns have been raised about the vaccine’s ability to spread COVID-19, but this is impossible because mRNA vaccines were not created with live SARSCoV-2 virus. Because it takes a few weeks for the body to develop T lymphocytes and B lymphocytes after vaccination, about 5.15 percent of the vaccinated individuals in the study still had the virus. Therefore, it is possible for someone to be infected with SARS-CoV-2 either before or just after receiving a vaccination and then become ill because there was not enough time for the vaccine to provide protection [16]. The literature acknowledges the effectiveness of SARS-CoV-2 vaccines in reducing disease severity, hospitalization, and mortality [26].

Rosenblum et al. [27] conducted a study on the safety data on 298 million doses of the mRNA COVID-19 vaccine administered in the first six months of the US vaccination program. The findings show that most reported adverse events were mild and short in duration. In another study, Barda et al. [28] evaluated the safety of the BNT162b2 mRNA vaccine with respect to a broad range of potential adverse events. The results revealed that the BNT162b2 vaccine was not associated with an elevated risk of most of the adverse events examined. Similarly, Thomas et al. [29] conducted a study on the safety and efficacy of the BNT162b2 mRNA COVID-19 vaccine and found that BNT162b2 had a favorable safety profile and was highly efficacious in preventing COVID-19.

## 5. Strength and Limitations

The strengths of our study include the fact that to date, extremely limited research exists in the literature comparing adverse effects with the severity of adverse effects of Pfizer-BioNTech, Oxford-AstraZeneca and Moderna, despite these being the most widely administered vaccines around the world. As the available literature is limited, we found it essential to conduct a study in order to gain thorough knowledge of the presenting adverse effects to determine which vaccine has the least adverse effects. The limitations of the study include a small sample size, given the circulation of the form’s online-based link self-administrated questionnaire. This is a cross-sectional study, based on self-reported adverse effects, which could be influenced by participants’ prior prejudice and misinformation about vaccines. The survey was completed by the public, so the adverse effects that occurred may be, at some level, overlooked.

## 6. Conclusions

All three vaccines, Pfizer-BioNTech, Oxford–AstraZeneca (ChAdOx1 CoV-19) and Moderna, caused post-vaccinal adverse effects. However, Pfizer-BioNTech COVID-19 vaccines caused significantly less adverse effects than Oxford–AstraZeneca (ChAdOx1) and Moderna. Females commonly reported more adverse events following COVID-19 vaccination compared to males. In addition, the group of people under the age of sixty was more likely than other age groups to experience adverse effects.

## 7. Study Implications

The present study provides valuable information on the adverse effects of three vaccines, Pfizer-BioNTech, Moderna and Oxford–AstraZeneca (ChAdOx1 CoV-19). There is a great need for such studies from various parts of the world for tracking the varying safety of vaccines, providing feedback to the public and policymakers, improving public trust in the safety of vaccines, and eradicating the stigma in society regarding the vaccine’s safety for combatting the COVID-19 pandemic. The present study results may enhance public confidence both at regional and international levels by dispelling misconceptions and conspiracy theories concerning the post-vaccination adverse effects of COVID-19 vaccines.

## Figures and Tables

**Table 1:** The demographic characteristics of the study participants (n = 426).

Variables	Frequency	Percent (%)
Gender Male Female	149 277	35 65
Age groups (years) 18–25 26–30 31–40 41–60 >60	113 43 93 149 28	26.5 10.1 21.8 35.0 6.6
Nationality Saudi Non-Saudi	401 25	94.1 5.9
Education Student higher secondary, undergraduate, and graduate Self employed Employed (government, private etc.)	226 91 109	53.1 21.4 25.6
Smoking History Smokers Non-smokers	64 362	15 85
Health status History of any chronic disease No history of any chronic disease	95 331	22.3 77.7

**Table 2:** Adverse effects of the first, second and third dose of Pfizer, Oxford–AstraZeneca, and Moderna COVID-19 vaccination (n = 426).

Parameters	First Doses Frequency (%)	Second Doses Frequency (%)	Third Doses Frequency (%)
Type of vaccine: Pfizer-BioNTech AstraZeneca (ChAdOx1) Moderna	333 (78.2%) 86 (20.2%) 7 (1.1%)	341 (80%) 68 (16%) 17 (4%)	339(79.6%) 19 (4.5%) 68 (16%)
Common adverse effects after vaccine: Feeling tired and headache Pain at the injection site Muscle and joint pain and feeling unwell Temperature and body shivering Dizziness Menstrual disorder No sides effect	187 (43.9%) 258 (60.6%) 138 (32.4%) 103 (24.2%) 54 (12.7%) 51 (12%) 82 (19.2%)	180 (42.3%) 238 (55.9%) 125 (29.3%) 101 (23.7%) 45 (10.6%) 41 (9.6%) 121 (28.4%)	155 (36.4%) 209 (49.1%) 106 (24.9%) 85 (20%) 44 (10.3%) 36 (8.5%) 152 (35.7%)
Feeling side effects after the vaccination First day Second day Third day No symptoms	147 (34.5%) 167 (39.2%) 11 (2.6%) 101 (23.7%)	143 (33.6%) 138 (32.4%) 11 (2.6%) 134 (31.5%)	136 (31.9%) 107 (25.1%) 8 (1.9%) 175 (41.1%)
The severity of the side effects Mild Moderate Sever No symptoms	102 (23.9%) 172 (40.4%) 55 (12.9%) 97 (22.8%)	88 (20.7%) 157 (36.9%) 55 (12.9%) 126 (29.6%)	82 (19.2%) 125 (29.3%) 59 (13.8%) 160 (37.6%)
How long adverse effect last after vaccination One-two days Three days Four or more No symptoms	210 (49.3%) 78 (20.4%) 36 (8.5%) 93 (21.8%)	195 (45.8%) 64 (15%) 38 (8.9%) 129 (30.3%)	158 (37.1%) 65 (15.3%) 37 (8.7%) 166 (39%)
Take medications Got any medication to reduce the severity of the side effects No taking medicine to reduce the severity of the side effects	40 (9.4%) 386 (90.6%)	222 (52.1%) 204 (47.9%)	139 (45.3%) 233 (54.7%)

**Table 3:** The rare adverse effects after Pfizer, Oxford–AstraZeneca, and Moderna COVID-19 vaccination (n = 426).

Low Side Effect	First Doses Frequency (%)	Second Doses Frequency (%)	Third Doses Frequency (%)
Vomiting	11 (2.6%)	5 (1.2%)	3 (0.7%)
Breathing congestion	7 (1.6%)	5 (1.2%)	11 (2.6%)
Skin itching or rash	18 (4.2%)	15 (3.5%)	10 (2.3%)
Drop-sugar level	4 (0.9%)	2 (0.5%)	2 (0.5%)

**Table 4:** Comparison of severity of adverse effects of Pfizer, Oxford–AstraZeneca, and Moderna COVID-19 vaccination between male and females.

Independent Samples *t*-Test						
Severity of Adverse Effects	Gender	N	Mean	Std. Deviation	T	*p* Value
Severity of the side effects after the first dose	Male	149	1.18	0.966	−3.974	0.000
	Female	277	1.57	0.963		
Severity of the side effects after the second dose	Male	149	1.08	1.017	−3.713	0.000
	Female	277	1.47	1.023		
Severity of the side effects after the third dose	Male	149	0.96	1.006	−3.406	0.000
	Female	277	1.32	1.114		

Significant symptoms are higher in females than males.

**Table 5:** Comparison of severity of adverse effects between Pfizer-BioNTech, Oxford-AstraZeneca and Moderna Vaccines.

Tests of Normality							
Severity of Adverse Effects	Type of the Vaccine	Kolmogorov-Smirnov			Shapiro-Wilk		
		Statistic	df	Sig.	Statistic	df	Sig.
Severity of the side effects after the first dose	Pfizer	0.246	333	0.000	0.858	333	0.000
	AstraZeneca Oxford (ChAdOx1)	0.272	86	0.000	0.835	86	0.000
	Moderna	0.357	7	0.007	0.787	7	0.030
Severity of the side effects after the second dose	Pfizer	0.239	341	0.000	0.852	341	0.000
	AstraZeneca Oxford (ChAdOx1)	0.267	68	0.000	0.822	68	0.000
	Moderna	0.229	17	0.018	0.858	17	0.014
Severity of the side effects after the third dose	Pfizer	0.232	339	0.000	0.838	339	0.000
	AstraZeneca Oxford (ChAdOx1)	0.304	19	0.000	0.783	19	0.001
	Moderna	0.270	68	0.000	0.799	68	0.000

**Table 6:** Comparison of severity of adverse effects among the participants who received Pfizer-BioNTech, Oxford-AstraZeneca and Moderna vaccines.

Severity of Adverse Effects	Type of the Vaccine	N	Mean Rank	Z	*p*-Value
Severity of the side effects after the first dose	Pfizer-BioNTech	333	198.36	25.597	0.001
	AstraZeneca Oxford (ChAdOx1)	86	266.38		
	Moderna	7	284.14		
	Total	426			
Severity of the side effects after the second dose	Pfizer-BioNTech	341	213.87	1.257	0.533
	AstraZeneca Oxford (ChAdOx1)	68	204.91		
	Moderna	17	240.44		
	Total	426			
Severity of the side effects after the third dose	Pfizer-BioNTech	339	213.15	0.018	0.991
	AstraZeneca Oxford (ChAdOx1)	19	216.24		
	Moderna	68	214.50		
	Total	426			

Adverse effects are significantly higher with Moderna then Oxford AstraZeneca and Pfizer-BioNTech.

## Data Availability

May be provided on reasonable request to the corresponding author.

## References

[1] Meo S.A., Al-Khlaiwi T., Usmani A.M., Meo A.S., Klonoff D.C., Hoang T.D. (2020). Biological and epidemiological trends in the prevalence and mortality due to outbreaks of novel coronavirus COVID-19. J. King Saud. Univ. Sci..

[2] World Health Organization WHO Coronavirus (COVID-19) Dashboard. https://covid19.who.int/.

[3] Da Rosa Mesquita R., Francelino Silva Junior L.C., Santos Santana F.M., Farias de Oliveira T., Campos Alcântara R., Monteiro Arnozo G., Rodrigues da Silva Filho E., Galdino Dos Santos A.G., Oliveira da Cunha E.J., Salgueiro de Aquino S.H. (2021). Clinical manifestations of COVID-19 in the general population: Systematic review. Wien. Klin. Wochenschr..

[4] Cazzolla A.P., Lovero R., Lo Muzio L., Testa N.F., Schirinzi A., Palmieri G., Pozzessere P., Procacci V., Di Comite M., Ciavarella D. (2020). Taste and Smell Disorders in COVID-19 Patients: Role of Interleukin-6. ACS Chem Neurosci..

[5] World Health Organization (WHO) (2022). Interim Recommendations for Use of the Inactivated COVID-19 Vaccine BIBP Developed by China National Biotec Group (CNBG), Sinopharm: Interim Guidance, First Issued the 7th of May 2021, Updated the 28th of October 2021, Updated the 15th of March 2022.

[6] Voysey M., Clemens S.A.C., Madhi S.A., Weckx L.Y., Folegatti P.M., Aley P.K., Angus B., Baillie V., Barnabas S., Bhorat Q. (2021). Safety and efficacy of the ChAdOx1 nCoV-19 vaccine (AZD1222) against SARS-CoV-2: An interim analysis of four randomised controlled trials in Brazil, South Africa, and the UK. Lancet.

[7] Esba L.C.A., Al Jeraisy M. (2021). Reported adverse effects following COVID-19 vaccination at a tertiary care hospital, focus on cerebral venous sinus thrombosis (CVST). Expert Rev. Vaccines.

[8] Saudi Food and Drug Authority. https://sfda.gov.sa/en/news/79059.

[9] Al Bahrani S., Albarrak A., Alghamdi O.A., Alghamdi M.A., Hakami F.H., Al Abaadi A.K., Alkhrashi S., Alghamdi M., Almershad M., Alenazi M.M. (2021). Safety and reactogenicity of the ChAdOx1 (AZD1222) COVID-19 vaccine in Saudi Arabia. Int. J. Infect. Dis..

[10] Alghamdi A.N., Alotaibi M.I., Alqahtani A.S., Al Aboud D., Abdel-Moneim A.S. (2021). BNT162b2 and ChAdOx1 SARS-CoV-2 post-vaccination side-effects among Saudi vaccinees. Front. Med..

[11] Alghamdi A., Ibrahim A., Alraey M., Alkazemi A., Alghamdi I., Alwarafi G. (2021). Side effects following COVID-19 vaccination: A cross-sectional survey with age-related outcomes in Saudi Arabia. J. Adv. Pharm. Educ. Res..

[12] Meo A.S., Masood A., Shabbir U., Ali H., Nadeem Z., Meo S.A., Alshahrani A.N., AlAnazi S., Al-Masri A.A., Al-Khlaiwi T. (2023). Adverse Effects of Sinopharm COVID-19 Vaccine among Vaccinated Medical Students and Health Care Workers. Vaccines.

[13] Centers for Disease Control and Prevention (CDC). Possible Side Effects After Getting a COVID-19 Vaccine. https://www.cdc.gov/coronavirus/2019-ncov/vaccines/expect/after.html.

[14] Darraj M.A., Al-Mekhlafi H.M. (2022). Prospective Evaluation of Side-Effects Following the First Dose of Oxford/AstraZeneca COVID-19 Vaccine among Healthcare Workers in Saudi Arabia. Vaccines.

[15] Raosoft Sample sie Calculator. http://www.raosoft.com/samplesize.html.

[16] Adam M., Gameraddin M., Alelyani M., Alshahrani M.Y., Gareeballah A., Ahmad I., Azzawi A., Komit B., Musa A. (2021). Evaluation of Post-Vaccination Symptoms of Two Common COVID-19 Vaccines Used in Abha, Aseer Region, Kingdom of Saudi Arabia. Patient Prefer. Adherence.

[17] Kurdee Z., Al-Shouli S., AlAfaleq N., Meo S.A., Alshahrani A., Alshehri A., Alkathiri N., Bin Saiedan S., Alzahrani Y. (2022). Public Perception towards the COVID-19 Vaccine in Riyadh, Saudi Arabia. Vaccines.

[18] El Zowalaty M.E., Järhult J.D. (2020). From SARS to COVID-19: A previously unknown SARS-related coronavirus (SARS-CoV-2) of pandemic potential infecting humans–Call for a One Health approach. One Health.

[19] Veiga V.C., Prats J.A.G.G., Farias D.L.C., Rosa R.G., Dourado L.K., Zampieri F.G., Machado F.R., Lopes R.D., Berwanger O., Azevedo L.C.P. (2021). Effect of tocilizumab on clinical outcomes at 15 days in patients with severe or critical coronavirus disease 2019: Randomised controlled trial. BMJ.

[20] Alamer E., Alhazmi A., Qasir N.A., Alamer R., Areeshi H., Gohal G., Qadri M., Hashem A., Algaissi A. (2021). Side Effects of COVID-19 Pfizer-BioNTech mRNA Vaccine in Children Aged 12–18 Years in Saudi Arabia. Vaccines.

[21] Andrzejczak-Grządko S., Czudy Z., Donderska M. (2021). Side effects after COVID-19 vaccinations among residents of Poland. Eur. Rev. Med. Pharmacol. Sci..

[22] Beattie M., Murphy D.J., Atherton I., Lauder W. (2015). Instruments to measure patient experience of healthcare quality in hospitals: A systematic review. Syst. Rev..

[23] Zhu F.-C., Li Y.-H., Guan X.-H., Hou L.-H., Wang W.-J., Li J.-X., Wu S.-P., Wang B.-S., Wang Z., Wang L. (2020). Safety, tolerability, and immunogenicity of a recombinant adenovirus type-5 vectored COVID-19 vaccine: A dose-escalation, open-label, non-randomised, first-in-human trial. Lancet.

[24] Julianne G., Marquez P., Su J., Calvert G., Liu R., Myers T., Nair N., Martin S., Clark T., Markowitz L. (2021). First month of COVID-19 vaccine safety monitoring-United States. US Dep. Health Hum. Serv. Dis. Control Prev..

[25] Alhazmi A., Alamer E., Daws D., Hakami M., Darraj M., Abdelwahab S., Maghfuri A., Algaissi A. (2021). Evaluation of Side Effects Associated with COVID-19 Vaccines in Saudi Arabia. Vaccines.

[26] Swan D.A., Bracis C., Janes H., Moore M., Matrajt L., Reeves D.B., Burns E., Donnell D., Cohen M.S., Schiffer J.T. (2021). COVID-19 vaccines that reduce symptoms but do not block infection need higher coverage and faster rollout to achieve population impact. Sci. Rep..

[27] Rosenblum H.G., Gee J., Liu R., Marquez P.L., Zhang B., Strid P., Abara W.E., McNeil M.M., Myers T.R., Hause A.M. (2022). Safety of mRNA vaccines administered during the initial 6 months of the US COVID-19 vaccination programme: An observational study of reports to the Vaccine Adverse Event Reporting System and v-safe. Lancet Infect. Dis..

[28] Barda N., Dagan N., Ben-Shlomo Y., Kepten E., Waxman J., Ohana R., Hernán M.A., Lipsitch M., Kohane I., Netzer D. (2021). Safety of the BNT162b2 mRNA Covid-19 Vaccine in a Nationwide Setting. N. Engl. J. Med..

[29] Thomas S.J., Moreira E.D., Kitchin N., Absalon J., Gurtman A., Lockhart S., Perez J.L., Pérez Marc G., Polack F.P., Zerbini C. (2021). Safety and Efficacy of the BNT162b2 mRNA Covid-19 Vaccine through 6 Months. N. Engl. J. Med..

